# Treatment strategies for inherited optic neuropathies: past, present and future

**DOI:** 10.1038/eye.2014.37

**Published:** 2014-03-07

**Authors:** P Yu-Wai-Man, M Votruba, A T Moore, P F Chinnery

**Affiliations:** 1 Wellcome Trust Centre for Mitochondrial Research, Institute of Genetic Medicine, Newcastle University, Newcastle upon Tyne, UK; 2 Departments of Neurology and Ophthalmology, Royal Victoria Infirmary, Newcastle upon Tyne, UK; 3 Moorfields Eye Hospital, London, UK; 4 NIHR Biomedical Research Centre, UCL Institute of Ophthalmology, University College London, London, UK; 5 School of Optometry and Vision Sciences, Cardiff University, Cardiff, UK; 6 Cardiff Eye Unit, University Hospital of Wales, Cardiff, UK

## Abstract

Bilateral visual loss secondary to inherited optic neuropathies is an important cause of registrable blindness among children and young adults. The two prototypal disorders seen in clinical practice are Leber hereditary optic neuropathy (LHON) and autosomal dominant optic atrophy (DOA). About 90% of LHON cases are due to one of three mitochondrial DNA (mtDNA) point mutations: m.3460G>A, m.11778G>A, and m.14484T>C, which affect critical complex I subunits of the mitochondrial respiratory chain. The majority of patients with DOA harbour pathogenic mutations within *OPA1*, a nuclear gene that codes for a multifunctional inner mitochondrial membrane protein. Despite their contrasting genetic basis, LHON and DOA share overlapping pathological and clinical features that serve to highlight the striking tissue-specific vulnerability of the retinal ganglion cell (RGC) layer to disturbed mitochondrial function. In addition to severe visual loss secondary to progressive optic nerve degeneration, a subgroup of patients will also develop a more aggressive syndromic phenotype marked by significant neurological deficits. The management of LHON and DOA remains largely supportive, but major advances in our understanding of the mechanisms underpinning RGC loss in these two disorders are paving the way for novel forms of treatment aimed at halting or reversing visual deterioration at different stages of the disease process. In addition to neuroprotective strategies for rescuing RGCs from irreversible cell death, innovative *in vitro* fertilisation techniques are providing the tantalising prospect of preventing the germline transmission of pathogenic mtDNA mutations, eradicating in so doing the risk of disease in future generations.

## Introduction

Hereditary optic nerve disorders result in significant chronic visual morbidity, and the minimum prevalence of affected individuals in the population has been estimated at 1 in 10 000.^1^ Leber hereditary optic neuropathy (LHON) and autosomal dominant optic atrophy (DOA) are the two classical paradigms for this group of disorders, and they account for most of the inherited optic atrophy cases seen in clinical practice. LHON is caused by mitochondrial DNA (mtDNA) point mutations, whereas, in DOA, the majority of cases are due to pathogenic mutations in the *OPA1* gene, which codes for an inner mitochondrial membrane protein.^1, 2^ Strikingly, both LHON and DOA share the same characteristic pathological features with selective degeneration of the retinal ganglion cell (RGC) layer, leading to progressive optic nerve degeneration and the onset of visual symptoms.^3^ Rarer autosomal recessive optic atrophy genes have also been identified and a common theme is emerging, with all of them so far encoding for key components of the mitochondrial machinery.^1, 2^ RGCs are therefore exquisitely sensitive to mitochondrial dysfunction, and the elucidation of the mechanisms involved is opening the way for therapeutic interventions targeting different stages of the disease process. In this review, recent advances in our understanding of LHON and DOA will be discussed, in addition to the practical management of this group of patients and emerging treatment options.

## Mitochondrial disorders

### Oxidative phosphorylation

Mitochondria are ubiquitous intracellular organelles present in all nucleated eukaryotic cells.^4, 5^ They form a highly interconnected network extending throughout the cell's structure and their overall morphology is dictated by the balance between mitochondrial fusion and fission. Mitochondria are bounded by two membranes and this arrangement creates two distinct anatomical compartments, namely, the intermembrane space and the internal matrix space. The mitochondrial respiratory chain consists of five multi-subunit complexes (I-V) embedded within the inner mitochondrial membrane, and it accounts for the bulk of the cell's adenosine triphosphate (ATP) production through the process of oxidative phosphorylation (OXPHOS).^4, 5^ In order to increase the effective surface area available for mitochondrial biogenesis, the inner mitochondrial membrane is thrown into multiple infoldings, known as cristae, and, unsurprisingly, aberrant cristae morphology is a frequent ultrastructural feature of disorders caused by mitochondrial dysfunction. OXPHOS is a tightly regulated process that involves the donation of high-energy electrons to complex I by the intermediate products (NADH and FADH_2_) generated from the beta-oxidation of fatty acids and glycolysis. As these electrons shuttle along the mitochondrial respiratory chain, a significant amount of energy is released and this is used to actively pump protons from the matrix compartment into the intermembrane space. Ubiquinone is a fat-soluble molecule present at a very high concentration within the inner mitochondrial membrane, and it has a unique role in OXPHOS as the only carrier that can efficiently transfer electrons from complexes I and II to complex III. The electrochemical proton gradient generated across the inner mitochondrial membrane is eventually harnessed by complex V (ATP synthase) for the conversion of ATP from adenosine diphosphate (ADP) and inorganic phosphate.

### Mitochondrial genetics

Mitochondria are unique in possessing their own genetic material in the form of a double-stranded circular DNA molecule about 16 569 base-pair long.^4, 5^ The mitochondrial genome is therefore very compact, but it codes for structural and functional components that are indispensable for normal mitochondrial function, including two ribosomal RNAs (12S and 16S rRNA), 22 transfer RNAs (tRNAs), and 13 polypeptide subunits of the mitochondrial respiratory chain complexes (I, III, V, and V). Another remarkable feature of mitochondrial genetics is its very high copy number with several thousands of mtDNA molecules being present in metabolically active cells such as neurons. Unlike nuclear DNA, mtDNA replicates continuously in dividing as well as in non-dividing cells, and this process occurs independently of the cell cycle. As a result of this relaxed replication machinery, a cell's total mtDNA content can be adjusted according to its bioenergetic requirements under both physiological and pathological conditions. Furthermore, the large number of mtDNA molecules present in each cell creates two possible genetic situations, referred to as homoplasmy or heteroplasmy. In the heteroplasmic state, two or more mtDNA variants are present at a specific nucleotide position, and this genetic admixture has been implicated in the variable disease expression that typifies mitochondrial disorders.^6^ Most pathogenic mtDNA mutations are heteroplasmic, and mitochondrial respiratory chain activity becomes compromised when the level of the mutant species exceeds a critical threshold (60–80%), which is both mutation and tissue specific.^4, 5^ In terms of genetic counselling, the mitochondrial genome shows strict maternal inheritance and male mutation carriers can be reassured that transmission to their own offspring will not occur. In contrast, all the children of a homoplasmic female carrier will harbour their mother's mtDNA mutation. The situation is rather more complex for a heteroplasmic mother as she could transmit a higher or a lower level of her mtDNA mutation to a particular offspring, which could influence the latter's risk of becoming clinically affected. The sometimes rapid shifts in mitochondrial allele frequencies that occur between maternally related generations have been explained by a ‘mitochondrial bottleneck' operating in the female germline.^7^

### Nuclear–mitochondrial interactions

Mitochondria have limited autonomy and they rely heavily on the nuclear genome for the vast majority of proteins required for mtDNA replication, transcription, and translation.^4, 5^ In 1988, two seminal papers were published linking for the first time specific mtDNA mutations with human disease: large-scale single mtDNA deletions in patients with mitochondrial myopathies and the m.11778G>A point mutation in families with LHON.^8, 9^ Families segregating classical mitochondrial phenotypes in a clear-cut Mendelian pattern of inheritance were subsequently reported, and the existence of nuclear genetic defects disrupting mitochondrial proteins was widely suspected. This hypothesis was confirmed in 2001, when mutations in the nuclear genes, *POLG* and *PEO1*, were identified in families with autosomal dominant chronic progressive external ophthalmoplegia (CPEO).^10^ The pathological hallmark of all these nuclear mitochondrial disorders is mtDNA instability, which can be *quantitative* in nature with a reduction in mtDNA copy number (depletion) or *qualitative* with the accumulation of high levels of somatic mtDNA mutations (predominantly deletions) in affected tissues. MtDNA molecules are located within the matrix compartment where they are packaged within intricate physical structures known as nucleoids.^11^ Although the components of these fundamental replicative units have not yet been fully established, both POLG and PEO1 are closely associated with mammalian nucleoids, highlighting the critical role played by these two proteins in mitochondrial genome maintenance. Mitochondrial transcription factor A (TFAM) has also been identified as a major nucleoid-organising protein responsible for packaging mtDNA and activating mitochondrial transcription.^10^ From a diagnostic perspective, the disruption of OXPHOS that ensues from the accumulation of secondary mtDNA abnormalities can be easily detected in skeletal muscle biopsies as cytochrome *c* oxidase (COX)-negative fibres.^6^ When present, these various molecular and histochemical features can be extremely helpful in the investigation of patients with suspected mitochondrial disease as they provide strong clues pointing towards an underlying nuclear genetic defect.

## Leber hereditary optic neuropathy

### Molecular genetics

**Figure 1:**
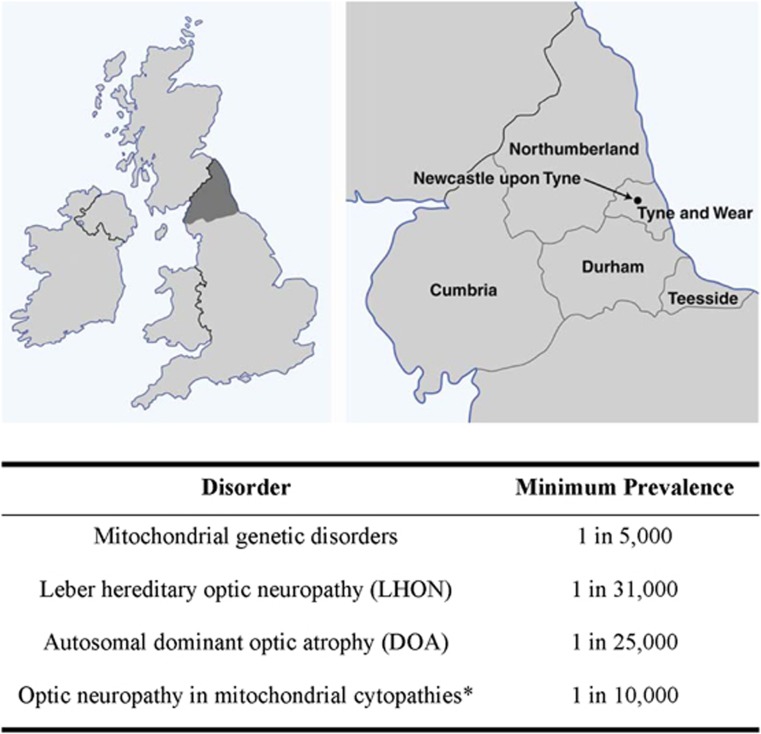
The prevalence of inherited optic neuropathies in the North of the United Kingdom. *Includes patients with LHON and DOA.^16^

LHON (OMIM 535000) is the most common of the primary mitochondrial DNA (mtDNA) disorders, and the minimum prevalence has been estimated at 1 in 31 000 in the North of the United Kingdom (Figure 1).^12^ Comparable prevalence figures of 1 in 39 000 and 1 in 50 000 have been reported in epidemiological studies from the Netherlands and Finland, respectively.^13, 14^ The majority of cases (90%) are due to one of three mtDNA point mutations: m.3460G>A (*MTND1*), m.11778G>A (*MTND4*), and m.14484T>C (*MTND6*), which affect key complex I subunits of the mitochondrial respiratory chain. The m.11778G>A mutation is by far the most common pathogenic mutation accounting for ∼70% of all LHON cases worldwide.^1, 2^ The one notable exception to this rule is the preponderance of the m.14484T>C mutation (∼ 90%) in patients of French Canadian descent secondary to a mutational founder event.^15^ In most diagnostic laboratories, screening for the three ‘primary' LHON mutations is carried out in the first instance in suspected cases. If this is negative and there is still a strong clinical suspicion of LHON, full mitochondrial genome sequencing can be considered. However, close liaison with an experienced laboratory is essential, not only because it remains a time consuming and expensive exercise, but interpretation of the sequencing results is not always straightforward.^16^ A number of relatively rare pathogenic mtDNA variants causing LHON have been confirmed, but others have only been reported in single patients or families and additional irrefutable proof of pathogenicity is still needed, which greatly complicates patient counselling (Table 1).

### Clinical features

Fundal abnormalities such as peripapillary telangiectatic vessels and transient retinal nerve fibre layer (RNFL) oedema can be observed in some asymptomatic LHON carriers.^17, 18^ On more detailed psychophysical testing, some unaffected LHON carriers also show subclinical optic nerve impairment with reduced colour vision and contrast sensitivity, and subnormal responses on multifocal electroretinography and visual evoked potentials.^19^ Disease conversion in LHON is characterised by acute or subacute, painless, central visual loss in one eye, followed weeks to months later by the fellow eye.^20^ The median inter-eye delay is 6–8 weeks, and the second eye is invariably affected within 1 year of disease onset. Bilateral simultaneous onset occurs in a proportion of patients (∼25%) with the caveat that some individuals are probably unaware that visual loss had been ongoing unnoticed in one eye before the second eye being involved. Although there are rare case reports of unilateral optic nerve involvement in LHON, it is important to exclude other underlying pathological causes in these highly atypical presentations. The peak age of onset is in the second and third decades of life, and it is unusual for LHON carriers to experience visual loss beyond the age of 50 years.^20^ Visual acuity worse than 6/60 is the norm at the nadir, and there is an associated dense central or centrocecal scotoma with profound dyschromatopsia. Interestingly, the pupillary light reflexes are relatively preserved in LHON due to the sparing of a special class of melanopsin-containing RGCs that seem more resistant to mitochondrial dysfunction.^21^

**Figure 2:**
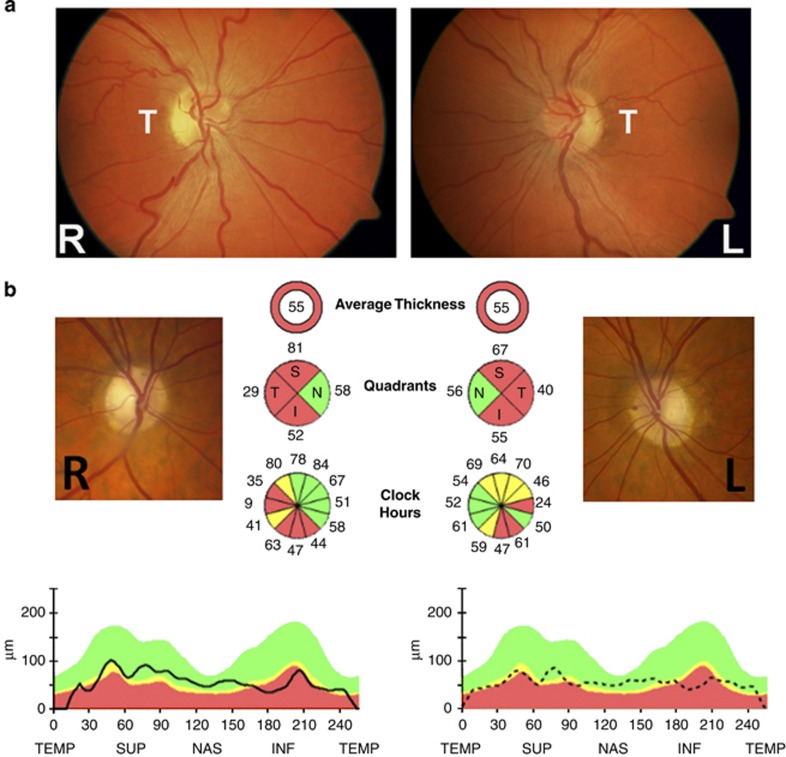
(a) Fundal abnormalities in Leber hereditary optic neuropathy. This m.11778G>A male carrier experienced visual loss in his right eye initially, followed 8 months later by his left eye. The fundus pictures were taken 1 month after the left eye had become affected. Best-corrected visual acuities at that point were counting fingers in the right eye and 6/36 in the left eye. There is temporal pallor of the right optic disc, whereas the left optic disc is still hyperaemic consistent with the more recent disease onset. Vascular tortuosity of the central retinal vessels can also be observed in both eyes. L, left eye; R, right eye; T, temporal papillomacular bundle. (b) Pattern of retinal ganglion cell loss in autosomal dominant optic atrophy. The patient harbours a pathogenic *OPA1* nonsense mutation within exon 27 (c.2713C>T, p.R905X). Pallor of the optic nerve head is more pronounced temporally and optical coherence tomography (OCT) imaging shows relative sparing of the nasal peripapillary retinal nerve fibre layer (RNFL). OCT measurements were obtained with the spectral-domain Cirrus platform (Carl Zeiss Meditec, Dublin, CA, USA). In addition to the average thickness, RNFL values are shown for each individual quadrant and each clock hour. The analysis software automatically selects the appropriate age-corrected normative range for the patient, and the RNFL measurements (dark traces) are represented within colour-coded distribution centiles: (i) red<1%, (ii) yellow 1–5%, and (iii) green 5–95%.

A careful dilated fundus examination can be particularly informative in the acute stage of LHON as patients classically exhibit a number of distinct abnormalities, namely, optic disc hyperaemia, peripapillary telangiectatic vessels, vascular tortuosity, and swelling of the retinal nerve fibre layer (RNFL) secondary to axoplasmic stasis (Figure 2a).^1, 2^ It is important to stress that the posterior pole can look entirely normal in a proportion (20–40%) of affected LHON carriers and the diagnosis may be delayed until optic disc pallor becomes apparent at around 6 weeks. This group of patients are frequently labelled as having functional visual loss, at least initially. In addition to pallor of the neuroretinal rim, non-glaucomatous cupping of the optic disc is also a recognised pathological feature of longstanding LHON cases, reflecting the progressive loss of RGCs that occurs in the chronic phase of the disease process.^1, 2^

The visual prognosis in LHON is poor and the majority of patients will remain legally blind with a significant detrimental impact on their overall quality of life.^22^ Spontaneous visual improvement can occur and it is most likely within the first year. Remarkably, it has also been reported several years or even decades following the initial visual loss. Visual recovery is usually heralded by the appearance of small islands of vision in the central visual field and, as the scotoma gets concurrently less dense, these fenestrations can greatly help with scanning vision and peripheral navigation.^1, 2^ LHON carriers harbouring the m.14484T>C mutation have the best visual prognosis with a partial visual recovery rate of 37–58%, compared with 4–25% for the m.11778G>A mutation.^12, 23, 24, 25^ Other reported positive prognostic factors for visual recovery in LHON include an earlier age of onset (<20 years), especially among young children; a subacute presentation with slow visual deterioration; and a relatively large optic disc.^26, 27^

### LHON plus phenotypes

An abiding mystery of LHON is the tissue-specific vulnerability of the RGC layer despite the ubiquitous presence of the pathogenic mtDNA mutation. Although visual loss is the defining feature of this mitochondrial disorder, cardiac conduction defects and neurological abnormalities such as ataxia, peripheral neuropathy, myopathy, and dystonia have been reported to be more common among LHON carriers compared with controls.^1, 2^ However, because of the relative rarity of these events, the causal link between the underlying mtDNA mutation and the development of these extraocular manifestations remains largely circumstantial. In 1992, Anita Harding and colleagues described a rather intriguing association between the m.11778G>A LHON mutation and a demyelinating syndrome that was clinically and radiologically indistinguishable from multiple sclerosis (MS).^28^ Harding disease, as this association became known subsequently, is more common among female LHON carriers and those harbouring the m.11778G>A mutation. However, additional functional work is needed to determine whether the mtDNA mutation is directly influencing the development of an MS-like illness in addition to visual failure, or whether this association simply represents a statistical chance occurrence.^29, 30^ In a small number of families from Holland, Australia, and North America, rare pathogenic mtDNA variants have been linked with particularly severe LHON plus phenotypes complicated by spastic dystonia, ataxia, juvenile-onset encephalopathy, and psychiatric disturbances.^1^

### Incomplete penetrance and sex bias

Not all LHON mutation carriers will experience visual loss during their lifetime. The conversion rate for male carriers is ∼50% compared with ∼10% for female carriers.^1^ LHON is therefore characterised by both marked incomplete penetrance and a striking male bias for visual loss. The secondary factors that modulate the phenotypic expression of the underlying pathogenic mtDNA mutation have been the subject of intense research over the past two decades. Although we do not yet have the full picture of what is proving to be a relatively complex disease, a number of genetic, hormonal, and environmental factors have been identified, some of which are particularly relevant to patient counselling and the development of drug treatments for LHON.

Heteroplasmy is thought to influence disease penetrance based on the observation that affected LHON carriers have mutation levels greater than 60% in peripheral blood leukocytes.^31^ However, incomplete penetrance is still observed among heteroplasmic carriers with mutation levels exceeding this nominal threshold, and the majority of LHON families (80–90%) harbour homoplasmic mtDNA mutations.^12^ Heteroplasmy is therefore not a major contributor to the incomplete penetrance pattern seen in LHON. Another factor that could be modulating the risk of visual loss in LHON is the mitochondrial genetic background on which the pathogenic mtDNA mutation is segregating. The mitochondrial genome is highly polymorphic, and, during human evolution, ancient mtDNA variants have gradually clustered together in specific combinations known as haplogroups.^4, 5^ Mitochondrial haplogroups have a differential effect on the efficiency of the mitochondrial respiratory chain, and this could possibly influence the overall deleterious impact of the LHON mutation on RGC survival.^32, 33^ In a large meta-analysis of Caucasian LHON pedigrees, haplogroup J was associated with a significantly increased risk of visual loss among m.11778G>A and m.14484T>C carriers, whereas m.3460G>A carriers were more likely to become affected on a haplogroup K background.^34^ LHON carriers with the m.11778G>A mutation had a lower risk for visual loss when the mutation arose on haplogroup H. Other haplogroup associations have been reported in families with LHON from mainland China, but none in those from South-East Asia.^35, 36^ The influence of specific mtDNA polymorphisms on the risk of visual failure in LHON is therefore not entirely clear-cut, and further investigation is warranted.

The predominance of affected males in LHON cannot be explained by mitochondrial inheritance. Segregation analysis of a large number of LHON families was consistent with the existence of a recessive X-linked susceptibility gene acting in synergy with the mtDNA mutation to precipitate visual loss.^37^ Three independent linkage studies have revealed overlapping candidate regions on the X-chromosome, but the actual modifier gene has still not yet been identified.^38, 39, 40^ The existence of autosomal nuclear modifiers in LHON remains a distinct possibility, and the situation could prove even more complex if different combinations of nuclear genes are segregating in different ethnic populations.

Another obvious explanation for the striking marked male bias in LHON is a protective benefit from female sex hormones. Interestingly, treatment with 17*β*-oestradiol resulted in reduced reactive oxygen species (ROS) levels, increased activity of the antioxidant enzyme superoxide dismutase (SOD), and more efficient mitochondrial biogenesis in LHON cybrid cell lines.^41^ As RGC cell bodies have a high concentration of oestrogen *β* receptors, supplementation with oestrogen-like compounds represents an attractive therapeutic option. Various environmental triggers have been implicated in precipitating the onset of visual loss in LHON, including head trauma, psychological stress, exposure to industrial toxins, and drugs with mitochondrial toxic effects such as antiretrovirals and ethambutol.^1, 2^ A recent multicentre study that enrolled 125 Northern European LHON pedigrees has provided important insight into the role of smoking and alcohol consumption on disease expression.^42^ Smoking was strongly associated with an increased risk of visual loss, and this detrimental effect was more marked for heavy smokers compared with light smokers. Various toxins present in cigarette smoke are capable of depressing mitochondrial ATP synthesis either through a direct inhibitory effect on complex I activity or by reducing arterial oxygen concentration. There was also a trend towards an increased risk of visual failure with heavy binge drinking, but this effect was not as marked as for smoking.

### Disease mechanisms

The papillomacular bundle is affected early and much more severely in LHON. The preferential involvement of the RGCs within the papillomacular bundle is likely related to their relatively small calibres and limited mitochondrial energetic reserves.^43^ Two major mechanisms have been proposed to precipitate RGC loss in LHON—a biochemical respiratory chain defect and increased levels of reactive oxygen species (ROS).^44^ There are supporting lines of evidence for both these pathological pathways, and they represent obvious therapeutic targets to halt the catastrophic loss of RGCs that occurs during the acute phase of LHON. The three most common primary LHON mutations (m.3460G>A, m.11778G>A, and m.14484T>C) all affect critical complex I subunits, and impaired complex I-driven ATP synthesis has been identified using both *in vitro* and *in vivo* biochemical assays.^1, 3^ A mouse model has recently been developed that replicates some of the key histopathological features seen in the optic nerves of affected LHON patients, namely the preferential degeneration of the smallest calibre RGC fibres, marked axonal swelling, and the accumulation of dysmorphic mitochondria within demyelinated segments.^45^ Increased ROS levels were observed with no reduction in ATP synthesis, strongly supporting oxidative stress as the major mechanism driving the loss of RGCs, at least in that specific mouse model.

## Autosomal dominant optic atrophy

### Molecular genetics

Autosomal dominant optic atrophy (DOA, OMIM 605290) is the most commonly diagnosed inherited optic neuropathy, and, in a recent epidemiological study, the minimum prevalence was estimated at 1 in 25 000 in the North of the United Kingdom (Figure 1).^46^ The majority of families with DOA (∼ 60%) harbour pathogenic mutations in *OPA1* (3q28-q29), which codes for a dynamin-related GTPase protein located within the inner mitochondrial membrane.^47, 48^ It is a ubiquitous protein and abundant levels have been detected in RGCs, the inner ear, and the brain. *OPA1* is highly polymorphic and over 200 pathogenic mutations have been identified with hotspots in the catalytic GTPase domain (exons 8–15) and the dynamin central domain (exons 16–23).^49^ Most pathogenic mutations result in a marked reduction in the level of the OPA1 protein, and the pathological significance of haploinsufficiency is further highlighted by those rare families with large-scale deletions spanning the entire *OPA1* coding region.^50^

### Clinical features

DOA has an insidious onset in early childhood and it typically presents with bilateral, symmetrical, central visual loss and dyschromatopsia. There is a wide variability in disease severity with visual acuities ranging from 6/6 to the detection of hand movement.^51^ Asymptomatic *OPA1* mutation carriers with subclinical disease are frequently identified during contact tracing. If there is no definite family history, it is therefore important to examine other family members of a patient with suspected inherited optic atrophy to establish more clearly the pattern of inheritance. Visual deterioration has been reported in 19 to 67% of patients with DOA on long-term follow-up, with 13 to 46% being registered legally blind.^1, 2^ Visual decline in DOA is usually slow, and a good proportion of patients will retain good functional vision late into their disease. However, the rate of visual loss can be highly variable both between and within families, and some patients with DOA experience a much faster rate of disease progression.^51^

Similar to LHON, the pathological hallmark of DOA is the preferential early loss of RGCs within the papillomacular bundle, accounting for the distinctive temporal wedge of pallor that is commonly observed at the optic nerve head (Figure 2b). However, pallor of the neuroretinal rim can be subtle, and the demonstration of pathological thinning of the retinal nerve fibre layer (RNFL), especially in the temporal quadrant, with optical coherence tomography (OCT) imaging can be particularly helpful.^52^ Electrophysiological testing can also be informative in these equivocal cases. The pattern visual evoked potentials in DOA is characterised by abnormally prolonged latencies and a variable decrease in magnitude. The N95 component of the pattern electroretinogram (pERG) is usually selectively depressed consistent with primary dysfunction of the RGC layer. Deep excavation of the optic disc with an enlarged cup-to-disc ratio greater than 0.5 is another typical morphological feature observed in DOA.^51^ It is therefore not surprising that patients with DOA are often misdiagnosed as having normal tension glaucoma, especially if the degree of optic disc pallor is mild and there is no convincing family history. Limited post mortem studies suggest the relative preservation of melanopsin-containing RGCs, and this could account for the lack of an afferent pupillary defect in patients with DOA, which is another clinical feature shared with LHON.^21^

### DOA plus phenotypes

Progressive central visual loss is the defining feature of DOA, but with greater access to molecular genetic testing, a much broader array of clinical presentations have now been linked to pathogenic *OPA1* mutations. The most common extraocular feature is sensorineural hearing loss developing in the first two decades of life and a specific *OPA1* mutation in exon 14 (c.1334G>A, p.R445H) has been strongly associated with the co-occurrence of deafness and optic atrophy.^53^ In a large multicentre study, nearly 20% of *OPA1* mutation carriers developed a more severe neuromuscular phenotype complicated by varying combinations of deafness, ataxia, myopathy, peripheral neuropathy, and CPEO.^53^ Interestingly, some patients have also been reported with clinical features indistinguishable from MS, hereditary spastic paraplegia (HSP), and the inherited spinocerebellar degenerations. From a mechanistic perspective, these so-called dominant optic atrophy plus (DOA+) phenotypes confirm that the pathological consequences of *OPA1* mutations are not limited to RGCs, but they clearly extend to other central neuronal populations, peripheral nerves, and skeletal muscle.

### Disease mechanisms

OPA1 is a multifunctional protein located within the inner mitochondrial membrane. It is a critical pro-fusion protein and pathogenic *OPA1* mutations result in marked mitochondrial network fragmentation.^54^ This physical disruption has a knock-on effect on the stability of the mitochondrial respiratory chain complexes, resulting in increased ROS levels and a reduction in overall ATP synthesis.^55, 56^ Pro-apoptotic cytochrome *c* molecules are carefully sequestered within the cristae spaces by the zipper-like action of OPA1 and mitochondria are major stores of calcium.^57, 58^ Mitochondrial network fragmentation results in the uncontrolled release of these potent pro-apoptotic factors, which ultimately trigger irreversible cell death. Although the mechanisms still need to be fully elucidated, *OPA1* mutations result in mitochondrial genome instability and the accumulation of multiple mtDNA deletions in affected tissues of some patients.^59^ Patients with DOA+ phenotypes harbour significantly higher levels of these somatic mtDNA abnormalities suggestive of a contributory role in the development of the more severe neuromuscular complications.^53^ Although OPA1 regulates a whole host of interrelated cellular pathways, compromised OXPHOS and elevated ROS levels are two major disease mechanisms that overlap with LHON, raising the exciting prospect of generic neuroprotective strategies applicable to both these mitochondrial optic neuropathies.

## Patient Management

### General supportive measures

The sudden onset of severe visual loss in LHON causes significant distress and anxiety for patients and their families. The majority of patients are young healthy adults, and the shock of receiving a genetic confirmation of LHON is often compounded by the diagnostic delays and uncertainties that are not uncommon for rare genetic disorders. Although DOA has a less marked presentation compared with LHON, visual loss can be progressive and in a quality of life survey, half of all the patients interviewed reported high levels of anxiety and depression.^60^ Clinicians therefore have an important role to play in facilitating access to rehabilitative services such as low visual aids and occupational therapy. Patient groups and information websites can also provide invaluable support, especially in the immediate period after a diagnosis has been made (https://sites.google.com/site/planetleeder/lhon and http://www.lhon.org/lhon/LHON.html, accessed on 8 December 2013). In addition to visual loss, patients with LHON and DOA can also develop neurological features such as ataxia, peripheral neuropathy, and hearing impairment. Clinicians need to be aware of these ‘plus' phenotypes to ensure their early detection, and patients in this high-risk group are best served by a multidisciplinary team to aggressively manage and minimise the functional consequences of these systemic complications.

### Genetic counselling

The mitochondrial genome is maternally inherited and male LHON carriers can be reassured that their children are not at risk of inheriting their genetic defect. In contrast, female carriers will transmit the mutation to all their children, but, if the mutation is heteroplasmic, the level of the mtDNA mutation transmitted can fluctuate, sometimes markedly, as a result of the mitochondrial genetic bottleneck. Prenatal testing for heteroplasmic female LHON carriers is therefore unhelpful because the mutant load detected in amniocytes and chorionic villi may not correspond to that in other fetal cell populations, especially those destined to mature into RGCs.

A frustrating aspect of the management of LHON is the current inability to accurately predict whether a familial carrier will eventually experience visual loss. Despite these limitations, individuals can be counselled based on the two major identifiable risk factors in this disorder, namely age and sex.^1, 2^ Male carriers have about a 50% lifetime risk of visual failure compared with only 10% for female carriers, and the peak age of onset is in the second and third decades of life. As a general health measure, patients with mitochondrial disorders should be strongly advised not to smoke and to moderate their alcohol intake. This is especially the case for unaffected LHON carriers as smoking, and to a lesser extent excessive binge drinking, has been linked with an increased risk of disease conversion.^42^ It also seems sensible to avoid exposure to other putative environmental triggers for visual loss in LHON, in particular industrial toxins and drugs with mitochondrial-toxic effects, for example ethambutol.^1, 2^

DOA is an autosomal dominant Mendelian disorder, and, although genetic counselling is straightforward, providing accurate information about likely disease severity is challenging for several reasons. It is not possible to predict the rate of progression of visual loss, which can be highly variable even within families harbouring the same pathogenic *OPA1* mutation.^51^ Furthermore, although patients harbouring missense *OPA1* mutations in the GTPase domain of the protein have a statistically higher risk of developing DOA+ phenotypes, not all of them will do so, and the development of neurological features only becomes manifest in mid- to late adulthood, introducing another element of uncertainty.^53^ *OPA1* mutation carriers have a 50% chance of transmitting the pathogenic allele to each of their children, and pregnant women can request prenatal testing after having been appropriately counselled. Prenatal diagnosis is possible by analysing DNA extracted from fetal cells and the method used will depend on the mother's personal choice and her gestational status, either amniocentesis (15–18 weeks) or chorionic villus sampling (10–12 weeks).

## Treatment options—LHON

### Mitochondrial cocktails

There is currently only limited evidence to support the use of any intervention in mitochondrial disorders.^61^ In a recent systematic review of the literature, only 35 out of 1039 publications on treatments for mitochondrial diseases included more than five patients, and most of these studies suffered from significant methodological weaknesses.^62^ Over the years, various combinations of vitamins (B_2_, B_3_, B_12_, C, E, and folic acid) and other compounds with putative mitochondrial antioxidant and bioenergetics properties have been used to treat patients with mitochondrial disease, including LHON, but none with any convincing proof of efficacy.^61, 62, 63^ The list of supplements that are frequently promoted on the internet includes alpha-lipoic acid, carnitine, creatine, and L-arginine.

### Ubiquinone analogues

Ubiquinone is an essential carrier that ensures the efficient transfer of electrons along the mitochondrial respiratory chain. Pathogenic mtDNA mutations associated with LHON destabilise complex I resulting in a reduction in ATP synthesis and increased ROS levels. An attractive strategy to restore the flow of electrons along the mitochondrial respiratory chain is to bypass the blockage occurring at the level of complex I by enabling and maximising the direct shuttling of electrons to complex III. Coenzyme Q10 (CoQ10) is a ubiquinone analogue with a long history of use for patients with a broad range of mitochondrial disorders.^61, 62, 63^ Although there is a sound scientific basis based on the potential antioxidant and bioenergetic properties of CoQ10, a Cochrane review found no objective evidence of any significant patient benefit in the limited number of small studies that have been published.^61^ Putting these methodological issues aside, the lack of efficacy of CoQ10 could be due to its lipophilic nature, this physical property hindering its delivery to mitochondria following oral administration of the drug. There is one anecdotal report of an affected patient carrying the m.11778G>A mutation who experienced marked visual improvement after he was started on CoQ10 about 8 months following the first onset of symptoms.^64^ This could simply have been a chance occurrence given that spontaneous visual improvement has been noted in up to 25% of patients harbouring this mtDNA mutation.

Idebenone and EPI-743 are newer generation shorter-chain analogues of ubiquinone that are reported to be more potent compared with CoQ10, at least *in vitro*.^28, 65^ The possible visual benefit of idebenone in LHON was first reported in 1992 by Mashima and colleagues who treated a 10-year-old boy harbouring the m.11778G>A with a relatively low daily dose of 90 mg/day.^66^ The obvious confounding factor in this case is the relatively young age of the patient, as childhood-onset LHON seems to be a distinct entity associated with a much more benign clinical course and a higher rate of spontaneous visual recovery.^26^ In a larger case series from the same Japanese group, 14 patients with LHON were treated with a combination of idebenone (180 mg/day), vitamin B_2_, and vitamin C.^67^ No significant difference in the proportion of eyes showing visual recovery was observed when this intervention group was retrospectively compared with 14 untreated patients. However, if it occurred, a faster rate of visual recovery was reported in the treated eyes compared with the untreated eyes. In contrast to these results, Barnils and colleagues^68^ found no visual benefit with the use of the same treatment cocktail in two visually affected individuals carrying the m.11778G>A mutation.

To address the true potential of idebenone as a treatment modality in LHON, a multicentre, double-blind, randomised placebo-controlled trial was initiated. RHODOS (Rescue of Hereditary Optic Disease Outpatient Study, ClinicalTrials.gov identifier: NCT00747487) enrolled 85 patients with a confirmed primary mtDNA mutation (m.3460G>A, m.11778G>A, and m.14484T>C) and with disease duration of up to 5 years.^28^ The active arm received idebenone (900 mg/day), and no adverse drug reactions occurred over a treatment period of 24 weeks. This trial failed to show benefit for its pre-specified primary end point (best recovery of visual acuity at week 24), but all of the secondary end points showed a positive trend towards visual improvement in the idebenone-treated group. A *post hoc* analysis of the trial data also indicated that patients with discordant visual acuities (LogMAR>0.2), and hence at highest risk of further deterioration in the least affected eye, were more likely to benefit from treatment with idebenone. In the follow-up study (RHODOS-OFU), the beneficial effect of 24 weeks of treatment with idebenone seemed to persist following discontinuation of the active medication at the end of the trial.^69^ In a large retrospective Italian study involving 103 patients with LHON, 44 patients with visual loss of 1 year's duration or less were treated with idebenone and followed for at least 5 years.^70^ A greater proportion of patients receiving idebenone recovered vision and the most consistent prognostic factors associated with visual recovery were the early initiation of treatment and a more prolonged course of treatment.

A marketing authorisation application by Santhera Pharmaceuticals Ltd (Liestal, Switzerland) to the European Medicines Agency's Committee for Medicinal Products for Human Use (CHMP) has recently received an unfavourable opinion and additional clinical evidence for the efficacy of idebenone in LHON has been requested by CHMP (http://www.santhera.com/index.php?docid=212&vid=&lang=en&newsdate=201301&newsid=1671337&newslang=en, accessed on 8 December 2013). The Newcastle Mitochondrial Research Group is planning a second double-blind, randomised, placebo-controlled trial that will investigate the benefit of treatment with a higher dose of idebenone (2250 mg/day) given for a total duration of 48 weeks instead of 24 weeks as for RHODOS (Table 2). Further work is also needed to determine the appropriate duration of treatment, once initiated, and whether there is a place for the treatment of asymptomatic carriers or patients with longstanding visual loss. Although idebenone is currently not licensed for clinical use, patients with LHON frequently opt to gain access to it at their own expense from various internet sources. EPI-743 has been used in an open-labelled study of five patients with LHON who were treated within 90 days of disease conversion. Although the results need to be confirmed in a properly designed clinical trial, four out of five patients demonstrated arrest of disease progression and variable extents of visual improvement.^65^

### Brimonidine

Brimonidine is a topical *α*-2 agonist that is commonly used to lower intraocular pressure in the management of glaucoma.^71^ Some studies have suggested that brimonidine can exert a synergistic RGC neuroprotective effect by upregulating a number of antiapoptotic factors and by blocking glutamate excitotoxicity induced by mitochondrial oxidative stress.^71^ Topical brimonidine has therefore been tested as a prophylactic agent for second-eye involvement in an open-labelled study of nine patients with unilateral acute visual loss secondary to LHON.^72^ Brimonidine failed to prevent fellow eye involvement, and there was no evidence of any visual benefit following the onset of visual loss. Although this study was negative, raised intraocular pressure has been linked with an increased risk of visual loss in LHON and an agent with putative neuroprotective properties such as brimonidine could represent an ideal choice of treatment for unaffected LHON carriers diagnosed with glaucoma or ocular hypertension.^73^

### Steroids and immunosuppressants

Patients with LHON are not infrequently treated with high-dose steroids before a molecular diagnosis has been secured to exclude the possibility of an inflammatory optic neuropathy. Steroids do not prevent the involvement of the fellow unaffected eye in LHON, and there has been no reported benefit on disease progression and the final visual outcome.^63^ In an *in vitro* experimental paradigm involving the supplementation of the culture media with hydrogen peroxide, LHON cybrids harbouring the m.11778G>A mutation showed an increased sensitivity to oxidative stress.^74^ This increased susceptibility to undergo apoptosis was postulated to be secondary to a toxic rise in intracellular calcium and the activation of the mitochondrial transition pore (MTP). Pre-treatment with cyclosporin A blunted the deleterious consequences of hydrogen peroxide by blocking the MTP pore, indicating a possible therapeutic pathway for LHON. The antiapoptotic effect of cyclosporine A has also been demonstrated in LHON cybrids harbouring the m.14484T>C and m.14279G>A mutations.^75^ On the basis of these *in vitro* data, a French study is currently underway recruiting patients with unilateral visual loss from LHON for treatment with cyclosporin A in an attempt to prevent the involvement of the fellow eye (Dr Dominique Bonneau, University of Angers, France, personal communication).

### Hyperbaric oxygen therapy

There is highly anecdotal ‘internet' evidence of patients with LHON benefiting from hyperbaric oxygen therapy (HBOT) (http://hyperbariclink.blogspot.co.uk/2012/06/in-news-hyperbaric-oxygen-therapy-for.html, accessed on 8 December 2013). The purported rationale for this treatment is to provide increased levels of oxygen to RGCs during the acute phase of LHON with the aim of improving mitochondrial biogenesis. HBOT is a controversial treatment modality that has been applied with limited success to other optic nerve disorders such as radiation-induced optic neuropathy and anterior ischaemic optic neuropathy.^76^ Given the rather slim evidence base, the theoretical toxic effects of supraphysiological levels of oxygen in LHON should be considered in the context of a dysfunctional mitochondrial respiratory chain that is already producing increased ROS levels.

### Near-infrared light therapy

Near-infrared light (NIR) therapy has been shown to improve mitochondrial function and cellular survival in various models of wound healing, neurodegeneration and methanol-induced retinal toxicity.^77^ Although these findings are not universally accepted and the mechanisms are not fully understood, NIR photobiomodulation is thought to increase ATP synthesis by stimulating the activity of cytochrome *c* oxidase (complex IV).^77^ The application of NIR therapy to RGCs via a light-emitting diode has therefore been proposed as a possible rescue strategy for LHON. A study was initiated to investigate the visual benefit of NIR therapy in affected LHON carriers, but it has been terminated because of the inability to record reliable pERG measurements, which was the planned primary outcome measure, due to poor subject fixation (http://clinicaltrials.gov/ct2/show/NCT01389817?term=LHON+whelan&rank=1, accessed on 8 December 2013).

## Treatment options: DOA

Compared with LHON, the rate of RGC loss in DOA is relatively slow and the detection of a clinically meaningful benefit over the course of a 1- or 2-year treatment trial is a major methodological consideration. Long-term natural history studies are therefore urgently needed to more precisely define the visual parameters that are most sensitive at detecting a significant change in optic nerve structure and function for insidiously progressive optic nerve disorders like DOA (Table 2). Idebenone has recently been evaluated in a limited case series involving seven patients with DOA and confirmed pathogenic *OPA1* mutations.^78^ A variable daily dose of idebenone was used (270–675 mg), and all the patients were treated and reviewed for at least 1 year. No adverse drug reactions were reported and some improvement in visual function was reported for five of the seven idebenone-treated patients. The results of this pilot study remain preliminary and a randomised, placebo-controlled trial with an adequate duration of follow-up will be needed to prospectively evaluate the possible benefit of using idebenone in DOA. Looking into the future, an open-labelled study of EPI-743 for patients with DOA is currently under preparation (Dr Valerio Carelli, University of Bologna, Italy, personal communication), and the potential benefit of near-infrared light in rescuing RGC loss is also being tested in a mouse model of DOA harbouring a splice site mutation (c.1065+5g>a) within intron 10 of the *Opa1* gene (Professor Marcela Votruba, Cardiff University, UK).^79^

## Future prospects and challenges

### Drug screening and development

With idebenone and EPI-743, we are witnessing the first tentative steps in our effort to try and modulate disease progression for mitochondrial optic neuropathies. Several research groups worldwide are actively pursuing the identification of novel neuroprotective agents for other, more prevalent, optic nerve disorders such as glaucoma and anterior ischaemic optic neuropathy, the results of which are likely to be highly relevant for LHON and DOA.^71, 80^ The technological revolution of the past decade has also launched a new era of personalised medicine with powerful complementary approaches that combine the *in vitro* screening of small molecule libraries with further *in vivo* validation using existing animal models. Greater collaboration with the pharmaceutical industry will be an important element of this major translational push for rare genetic disorders. More than ever, clinical academics will have an important role to play in taking the lead and setting the broad directions for the clinical evaluation and validation of new drugs for inherited optic neuropathies. Some obvious candidates are compounds that are thought to activate mitochondrial biogenesis, for example oestrogen-like molecules and acetyl-L-carnitine (ALCAR), which is derived from the acetylation of L-carnitine within mitochondria. ALCAR has the advantageous property of crossing the blood–brain barrier, and this molecule promotes OXPHOS by upregulating the transcript levels of ‘master' genes involved in mitochondrial biogenesis, namely PGC-1*α*, PGC-1*β*, and TFAM.^81, 82^ A study is currently nearing completion looking at the effect of ALCAR on neuronal conduction along the visual pathways in patients with chronic LHON and disease duration of more than 2 years (http://apps.who.int/trialsearch/trial.aspx?trialid=EUCTR2009-016982-26-IT, accessed on 8 December 2013).

### Gene therapy

Gene replacement therapy for LHON is an attractive strategy, given the easy anatomical accessibility of the RGC layer for direct manipulation. However, the double-membrane nature of mitochondria presents a formidable series of technical challenges that need to be overcome. First and foremost, a highly efficient vector is needed to penetrate the relatively impermeable inner mitochondrial membrane and to allow a sufficient number of mitochondria to be transfected in order to achieve the desired gene replacement effect. A possible solution is to bypass the mitochondrial genome altogether by using an elegant alternative approach that relies on the nuclear allotopic expression of the gene of interest.^83^ The relevant mtDNA gene can be efficiently transfected into the nuclear compartment with an adeno-associated virus (AAV) vector after it has been reconfigured to fit the slightly different coding system operating within the nuclear genome. This hybrid nuclear-encoded protein has also been engineered with a specific targeting sequence to facilitate its efficient import into mitochondria, thereby compensating for the missing or dysfunctional mitochondrial protein. The potential of this gene therapy approach in rescuing the disease phenotype was first demonstrated in m.11778G>A LHON cybrids.^84^ The ability to rescue RGCs and improve visual function was subsequently confirmed *in vivo* by two independent research groups working on LHON rodent models expressing mutated ND4 (m.11778G>A) complex I subunits.^85, 86^ These groundbreaking experiments are paving the way for more advanced studies involving primates and ultimately patients with LHON (http://www.gensight-biologics.com/, accessed on 8 December 2013). However, a note of caution is required here given the ongoing debate as to whether the imported wild-type ND4 subunit actually integrates into the native complex I to produce a stable functional unit within the inner mitochondrial membrane.^1^

Proof-of-principle has also been demonstrated for two other forms of gene therapy based on nuclear allotopic expression. One strategy involves the transfection of the nuclear genome with the neuroprotective *SOD2* gene packaged into an AAV vector.^87^ The overexpression of the superoxide dismutase ROS scavenger in m.11778G>A LHON cybrids suppressed apoptosis and resulted in increased cell survival.^87^ The consolidation of the cell's antioxidant defences could therefore prove a complementary approach to the replacement of the mutated gene product in LHON. A radically different strategy is based on the xenotopic expression of Ndi1, an alternative NADH oxidase expressed in yeast (*Saccharomyces cerevisiae*) mitochondria. Ndi1 is a versatile enzyme that can bypass a malfunctioning complex I to restore downstream electron transfer while at the same time suppressing ROS overproduction.^88^ Successful rescue of optic nerve degeneration was achieved using the yeast *Ndi1* gene in a rat model of LHON that was generated by the injection of rotenone-loaded microspheres into the optic layer of the rat superior colliculus.^88^

Despite the technical difficulties of directly introducing genetic material into mitochondria, two recent studies have suggested that this could be achieved using two very different genetic engineering approaches. In one study, whole circular mtDNA molecules isolated from healthy human donors were complexed with human recombinant TFAM, and this genetic construct was able to gain direct entry into cells. When applied to m.11778G>A LHON cybrids, this novel strategy was able to restore cellular respiration and ATP synthesis by promoting mitochondrial biogenesis.^89^ In the other study, a mitochondrial targeting sequence was attached to the capsid element of an AAV vector carrying the replacement *ND4* subunit gene.^90^ This additional tag allowed the direct introduction of the wild-type *ND4* gene into the mitochondrial compartment, leading to the successful rescue of the pathological phenotype in both m.11778G>A LHON cybrids and a mouse model manifesting optic atrophy secondary to allotopic expression of the human *ND4* mutation.^90^ The introduced *ND4* gene construct is thought to remain episomal, which should prevent the mutagenic disruption of the endogenous genes contained within the host mitochondrial genome.^91^ Although direct mitochondrial gene delivery offers exciting therapeutic potential, these potentially groundbreaking findings should be viewed as preliminary until they have been independently replicated by other research groups.

Gene therapy for DOA is being contemplated for affected patients harbouring truncating *OPA1* mutations that result in haploinsufficiency. The strategy being developed involves the intravitreal injection of an AAV vector to deliver the wild-type *OPA1* gene to surviving RGCs in a mouse model of DOA carrying the exon 27 (c.2708-2711delTTAG) mutation.^92^ Although conceptually simpler than mitochondrial gene therapy, further methodological refinements are needed to achieve stable transfection in a sufficient proportion of RGCs to result in a clinically meaningful effect (Dr Guy Lenaers, University of Montpelier, France, personal communication). It is important to emphasise that all the gene therapy approaches being envisaged for LHON and DOA are still at an early experimental stage of development, and further evidence of their safety and efficacy is needed before the initiation of human trials can be contemplated.

### Stem cells

The therapeutic potential of stem cells is being investigated for a wide range of genetic eye disorders, and patients with mitochondrial optic neuropathies will frequently enquire with their clinicians whether they are likely to benefit from this form of treatment.^93^ Various poorly monitored ‘stem cell institutes' worldwide are promoting the use of non-validated experimental protocols, and patients with LHON and DOA need to be carefully advised before embarking on multiple expensive courses of treatment with the possible associated biological risks. In sharp contrast to this unregulated parallel market, there are a number of well-established research programmes that are rigorously assessing the possible application of stem cell technology for optic nerve disorders.^93, 94^ Two main paradigms are being explored, namely the generation and transplantation of RGCs, or the use of specific stem cell populations to generate trophic factors that promote RGC survival.^93, 94^ The technique for generating and purifying mature RGCs from embryonic stem cells or induced pluripotent stem cells is still in its infancy. Another major complicating factor is how to integrate these differentiated RGCs into the retina and then force them to make the right topographical connection. At least in the short term, it is more likely that human-derived RGCs will provide the ideal tool for drug screening and understanding the basic mechanisms regulating RGC physiology in both health and disease states.^71, 95^

A proportion of patients with LHON will experience partial visual recovery, mostly within 1 year of being affected, but sometimes several years after the onset of the disease. It is therefore highly likely that a subpopulation of RGCs that survived the initial massive wave of apoptosis remain in a state of suspended animation awaiting two possible fates: a final push towards apoptosis or a gradual recovery of cellular function. These surviving RGCs are being targeted by oral neuroprotective agents, but mesenchymal stem cells are also an interesting modality based on promising data from experimental glaucoma and optic neuritis studies.^71, 95^ Autologous mesenchymal stem cells can be isolated from bone marrow isolates, and they secrete several diffusible neurotrophic factors that are thought to promote neuronal survival in adverse metabolic and excitotoxic environments. Rather encouragingly, the injection of mesenchymal stem cells was well tolerated in a small proof-of-concept study of 10 patients with secondary progressive MS.^96^ Although not definitive, there was a suggestion of structural, functional, and physiological improvement in some visual end points suggestive of a neuroprotective effect. If confirmed, a similar strategy is entirely conceivable to halt or slow down the irreversible loss of RGCs in LHON and DOA.

### Prevention of germline transmission

*In vitro* fertilisation (IVF) techniques are currently being developed to prevent female carriers of childbearing age from transmitting pathogenic mtDNA mutations to the next generation. Two different IVF options have been proposed, namely pronuclear transfer and metaphase II spindle transfer, to generate the so-called ‘three-parent embryos' devoid of mutant mtDNA molecules.^97^ The aim of both techniques is to transfer the parental nuclear genetic material (devoid of the mother's mutant mtDNA molecules) into a donor cytoplast containing a normal wild-type mtDNA population. In the pronuclear technique, the male and female pronuclei are transferred post fertilisation into a mitochondrial donor zygote harbouring wild-type mtDNA.^98^ The alternative approach involves the transplantation of metaphase II spindles between unfertilised oocytes followed by intracytoplasmic sperm injection fertilisation.^99^ There seems to be no significant carryover of mutant mtDNA with both IVF approaches, and further work is currently underway in higher primates.^98, 100^ Preventing the germline transmission of pathogenic mtDNA mutations is an exciting development, but a stringent series of safety tests will first need to be fulfilled. A significant proportion of manipulated zygotes showed abnormal development and other iatrogenic genetic consequences must be considered, including an increased risk of aneuploidy and epigenetic abnormalities.^97^ The manipulation of human embryos also entails a number of important legal, religious, and ethical ramifications that will need to be addressed. The United Kingdom has been at the forefront of this important debate and an expert panel was recently convened by the Human Fertilisation and Embryology Authority (HFEA) to review the fertilisation methods that could be used to prevent mitochondrial disease. This public consultation indicated positive support for mitochondria replacement to take place, but subject to strict safeguards and careful regulation being adhered to (https://www.gov.uk/government/news/innovative-genetic-treatment-to-prevent-mitochondrial-disease, accessed on 8 December 2013).

## Conclusion

Translational research for mitochondrial optic neuropathies is entering an exciting accelerated phase providing renewed hopes to patients and their families that the inexorable visual loss associated with this group of disorders can at least be halted. We still need a better understanding of the pathological mechanisms underpinning the preferential vulnerability of RGCs in LHON and DOA, but these more basic studies are now proceeding in parallel with major national and transnational initiatives aimed at drug development and early phase clinical trials. The establishment of patient cohorts will provide a strong base to launch properly designed randomised, placebo-controlled trials and the prospective collection of long-term natural history data will be essential to identify the best outcome measures to detect a treatment benefit. The challenges of identifying and validating treatments for relatively rare inherited optic nerve disorders remain daunting, but 25 years after the birth of mitochondrial genetic medicine, tractable solutions are finally emerging.

## Figures and Tables

**Table 1:** Mitochondrial DNA variants identified in patients with Leber hereditary optic neuropathy

	*Mitochondrial gene*	*Nucleotide change*
Common variants (∼ 90%)	*MTND1*	m.3460G>A^a^
	*MTND4*	m.11778G>A^a^
	*MTND6*	m.14484T>C^a^
		
Rare variants (∼ 10%)	*MTND1*	m.3376G>A, m.3635G>A^a^, m.3697G>A, m.3700G>A^a^, m.3733G>A^a^, m.4025C>T, m.4160T>C, m.4171C>A^a^
	*MTND2*	m.4640C>A, m.5244G>A
	*MTND3*	m.10237T>C
	*MTND4*	m.11696G>A, m.11253T>C
	*MTND4L*	m.10663T>C^a^
	*MTND5*	m.12811T>C, m.12848C>T, m.13637A>G, m.13730G>A
	*MTND6*	m.14325T>C, m.14568C>T, m.14459G>A^a^, m.14729G>A, m.14482C>A^a^, m.14482C>G^a^, m.14495A>G^a^, m.14498C>T, m.14568C>T^a^, m.14596A>T
	*MTATP6*	m.9101T>C
	*MTCO3*	m.9804G>A
	*MTCYB*	m.14831G>A

a These mtDNA variants are definitely pathogenic.

They have been identified in ≥2 independent LHON pedigrees and show segregation with affected disease status.

The remaining putative LHON mutations have been found in singleton cases or in a single family, and additional evidence is required before pathogenicity can be irrefutably ascribed.^1^

**Table 2:** Translational research initiatives for inherited optic neuropathies

The National Institute for Health Research (NIHR) is spearheading an ambitious translational research programme for rare genetic diseases in the United Kingdom. Inherited optic neuropathies have been selected as one of the priority disease themes. As part of this specific NIHR study, we will undertake deep phenotyping of patients with LHON and DOA to define the natural history of the visual loss and to identify the most sensitive parameters for monitoring disease progression. Importantly, this study will provide useful information on the visual outcome measures that are most likely to reveal a clinically meaningful treatment effect in future clinical trials for pipeline neuroprotective agents. Please get in touch with us if you have eligible patients who could be enrolled into this national initiative for translational research into inherited optic neuropathies.

## References

[bib1] Yu-Wai-Man P Griffiths PG Chinnery PF Mitochondrial optic neuropathies—Disease mechanisms and therapeutic strategies Prog Retinal Eye Res 2011 30 (2 81 114 10.1016/j.preteyeres.2010.11.002 PMC3081075 21112411

[bib2] Fraser JA Biousse V Newman NJ The Neuro-ophthalmology of mitochondrial disease Survey Ophthalmol 2010 55 (4 299 334 10.1016/j.survophthal.2009.10.002 PMC2989385 20471050

[bib3] Carelli V Ross-Cisneros FN Sadun AA Mitochondrial dysfunction as a cause of optic neuropathies Prog Retinal Eye Res 2004 23 (1 53 89 10.1016/j.preteyeres.2003.10.003 14766317

[bib4] DiMauro S Schon EA Mechanisms of disease: Mitochondrial respiratory-chain diseases New Engl J Med 2003 348 (26 2656 2668 12826641 10.1056/NEJMra022567

[bib5] Schapira AHV Mitochondrial diseases Lancet 2012 379 (9828 1825 1834 22482939 10.1016/S0140-6736(11)61305-6

[bib6] McFarland R Taylor RW Turnbull DM A neurological perspective on mitochondrial disease Lancet Neurol 2010 9 (8 829 840 20650404 10.1016/S1474-4422(10)70116-2

[bib7] Cree LM Samuels DC Lopes SCdS Rajasimha HK Wonnapinij P Mann JR A reduction of mitochondrial DNA molecules during embryogenesis explains the rapid segregation of genotypes Nat Genet 2008 40 (2 249 254 18223651 10.1038/ng.2007.63

[bib8] Holt IJ Harding AE Morganhughes JA Deletions of Muscle Mitochondrial-DNA in Patients with Mitochondrial Myopathies Nature 1988 331 (6158 717 719 2830540 10.1038/331717a0

[bib9] Wallace DC Singh G Lott MT Hodge JA Schurr TG Lezza AMS Mitochondrial-DNA Mutation Associated with Lebers Hereditary Optic Neuropathy Science 1988 242 (4884 1427 1430 3201231 10.1126/science.3201231

[bib10] Hudson G Chinnery PF Mitochondrial DNA polymerase-gamma and human disease Hum Mol Genet 2006 15 R244 R252 16987890 10.1093/hmg/ddl233

[bib11] Holt IJ He J Mao C-C Boyd-Kirkup JD Martinsson P Sembongi H Mammalian mitochondrial nucleoids: organizing an independently minded genome Mitochondrion 2007 7 (5 311 321 17698423 10.1016/j.mito.2007.06.004

[bib12] Man PYW Griffiths PG Brown DT Howell N Turnbull DM Chinnery PF The epidemiology of Leber hereditary optic neuropathy in the North East of England Am J Hum Genet 2003 72 (2 333 339 12518276 10.1086/346066 PMC379226

[bib13] Spruijt L Kolbach DN de Coo RF Plomp AS Bauer NJ Smeets HJ Influence of mutation type on clinical expression of Leber hereditary optic neuropathy Am J Ophthalmol 2006 141 (4 676 682 16564802 10.1016/j.ajo.2005.11.007

[bib14] Puomila A Hamalainen P Kivioja S Savontaus M-L Koivumaki S Huoponen K Epidemiology and penetrance of Leber hereditary optic neuropathy in Finland Eur J Hum Genet 2007 15 (10 1079 1089 17406640 10.1038/sj.ejhg.5201828

[bib15] Macmillan C Johns TA Fu K Shoubridge EA Predominance of the T14484C mutation in French-Canadian families with Leber hereditary optic neuropathy is due to a founder effect Am J Hum Genet 2000 66 (1 332 335 10631164 10.1086/302716 PMC1288340

[bib16] Sitarz KS Chinnery PF Yu-Wai-Man P Disorders of the optic nerve in mitochondrial cytopathies: new ideas on pathogenesis and therapeutic targets Curr Neurol Neurosci Rep 2012 12 (3 308 317 22392506 10.1007/s11910-012-0260-0 PMC3342502

[bib17] Nikoskelainen E Ophthalmological findings in leber hereditary optic neuropathy, with special reference to the mtDNA mutations (vol 103, pg 504, 1996) Ophthalmology 1996 103 (7 998 10.1016/s0161-6420(96)30665-9 8600429

[bib18] Savini G Barboni P Valentino ML Montagna P Cortelli P De Negri AM Retinal nerve fiber layer evaluation by optical coherence tomography in unaffected carriers with Leber's hereditary optic neuropathy mutations Ophthalmology 2005 112 (1 127 131 15629832 10.1016/j.ophtha.2004.09.033

[bib19] Sadun AA Salomao SR Berezovsky A Sadun F Denegri AM Quiros PA Subclinical carriers and conversions in Leber hereditary optic neuropathy: a prospective psychophysical study Transact Am Ophthalmol Soc 2006 104 51 61 PMC1809912 17471325

[bib20] Yu-Wai-Man P Griffiths PG Hudson G Chinnery PF Inherited mitochondrial optic neuropathies J Med Genet 2009 46 (3 145 158 19001017 10.1136/jmg.2007.054270 PMC2643051

[bib21] La Morgia C Ross-Cisneros FN Sadun AA Hannibal J Munarini A Mantovani V Melanopsin retinal ganglion cells are resistant to neurodegeneration in mitochondrial optic neuropathies Brain 2010 133 2426 2438 20659957 10.1093/brain/awq155 PMC3139936

[bib22] Kirkman MA Korsten A Leonhardt M Dimitriadis K De Coo IF Klopstock T Quality of Life in Patients with Leber Hereditary Optic Neuropathy Invest Ophthalmol Vis Sci 2009 50 (7 3112 3115 19255150 10.1167/iovs.08-3166

[bib23] Harding AE Sweeney MG Govan GG Riordaneva P Pedigree Analysis in Leber Hereditary Optic Neuropathy Families with a Pathogenic Mtdna Mutation Am J Hum Genet 1995 57 (1 77 86 7611298 PMC1801226

[bib24] Macmillan C Kirkham T Fu K Allison V Andermann E Chitayat D Pedigree analysis of French Canadian families with T14484C Leber's hereditary optic neuropathy Neurology 1998 50 (2 417 422 9484365 10.1212/wnl.50.2.417

[bib25] Johns DR Heher KL Miller NR Smith KH Lebers hereditary optic neuropathy—clinical manifestations of the 14484 MUTATION Arch Ophthalmol 1993 111 (4 495 498 8470982 10.1001/archopht.1993.01090040087038

[bib26] Barboni P Savini G Valentino ML La Morgia C Bellusci C De Negri AM Leber's hereditary optic neuropathy with childhood onset Invest Ophthalmol Vis Sci 2006 47 (12 5303 5309 17122117 10.1167/iovs.06-0520

[bib27] Ramos CdVF Bellusci C Savini G Carbonelli M Berezovsky A Tamaki C Association of optic disc size with development and prognosis of Leber's hereditary optic neuropathy Invest Ophthalmol Vis Sci 2009 50 (4 1666 1674 19098324 10.1167/iovs.08-2695

[bib28] Harding AE Sweeney MG Miller DH Mumford CJ Kellarwood H Menard D Occurrence of a multiple sclerosis-like illness in women who have a Lebers hereditary optic neuropathy mitochondrial-dna mutation Brain 1992 115 (4 979 989 1393514 10.1093/brain/115.4.979

[bib29] Palace J Multiple sclerosis associated with Leber's hereditary optic neuropathy J Neurol Sci 2009 286 (1-2 24 27 19800080 10.1016/j.jns.2009.09.009

[bib30] Pfeffer G Burke A Yu-Wai-Man P Compston DA Chinnery PF Clinical features of MS associated with Leber hereditary optic neuropathy mtDNA mutations Neurology 2013 81 (24 2073 2081 24198293 10.1212/01.wnl.0000437308.22603.43 PMC3863351

[bib31] Chinnery PF Andrews RM Turnbull DM Howell N Leber hereditary optic neuropathy: Does heteroplasmy influence the inheritance and expression of the G11778A mitochondrial DNA mutation Am J Med Genet 2001 98 (3 235 243 11169561 10.1002/1096-8628(20010122)98:3<235::aid-ajmg1086>3.0.co;2-o

[bib32] Gomez-Duran A Pacheu-Grau D Lopez-Gallardo E Diez-Sanchez C Montoya J Lopez-Perez MJ Unmasking the causes of multifactorial disorders: OXPHOS differences between mitochondrial haplogroups Hum Mol Genet 2010 19 (17 3343 3353 20566709 10.1093/hmg/ddq246

[bib33] Gomez-Duran A Pacheu-Grau D Martinez-Romero I Lopez-Gallardo E Lopez-Perez MJ Montoya J Oxidative phosphorylation differences between mitochondrial DNA haplogroups modify the risk of Leber's hereditary optic neuropathy Biochim Biophys Acta 2012 1822 (8 1216 1222 22561905 10.1016/j.bbadis.2012.04.014

[bib34] Hudson G Carelli V Spruijt L Gerards M Mowbray C Achilli A Clinical expression of Leber hereditary optic neuropathy is affected by the mitochondrial DNA-haplogroup background Am J Hum Genet 2007 81 (2 228 233 17668373 10.1086/519394 PMC1950812

[bib35] Ji Y Zhang AM Jia X Zhang Y-P Xiao X Li S Mitochondrial DNA Haplogroups M7b1 ' 2 and M8a affect clinical expression of Leber hereditary optic neuropathy in Chinese families with the m.11778G -> A mutation Am J Hum Genet 2008 83 (6 760 768 19026397 10.1016/j.ajhg.2008.11.002 PMC2668067

[bib36] Tharaphan P Chuenkongkaew WL Luangtrakool K Sanpachudayan T Suktitipat B Suphavilai R Mitochondrial DNA haplogroup distribution in pedigrees of Southeast Asian G11778A Leber hereditary optic neuropathy J Neuro-Ophthalmol 2006 26 (4 264 267 10.1097/01.wno.0000249318.88991.c4 17204919

[bib37] Bu XD Rotter JI X-chromosome-linked and mitochondrial gene-control of Leber hereditary optic neuropathy—evidence from segregation analysis for dependence on X-chromosome inactivation Proc Natl Acad Sci USA 1991 88 (18 8198 8202 1896469 10.1073/pnas.88.18.8198 PMC52474

[bib38] Hudson G Keers S Man PYW Griffiths P Huoponen K Savontaus ML Identification of an X-chromosomal locus and haplotype modulating the phenotype of a mitochondrial DNA disorder Am J Hum Genet 2005 77 (6 1086 1091 16380918 10.1086/498176 PMC1285165

[bib39] Shankar SP Fingert JH Carelli V Valentino ML King TM Daiger SP Evidence for a novel x-linked modifier locus for leber hereditary optic neuropathy Ophthalmic Genet 2008 29 (1 17 24 18363168 10.1080/13816810701867607

[bib40] Ji Y Jia X Li S Xiao X Guo X Zhang Q Evaluation of the X-linked modifier loci for Leber hereditary optic neuropathy with the G11778A mutation in Chinese Mol Vision 2010 16 (47 416 424 PMC2838738 20300564

[bib41] Giordano C Montopoli M Perli E Orlandi M Fantin M Ross-Cisneros FN Oestrogens ameliorate mitochondrial dysfunction in Leber's hereditary optic neuropathy Brain 2011 134 220 234 20943885 10.1093/brain/awq276 PMC3025718

[bib42] Kirkman MA Yu-Wai-Man P Korsten A Leonhardt M Dimitriadis K De Coo IF Gene-environment interactions in Leber hereditary optic neuropathy Brain 2009 132 2317 2326 19525327 10.1093/brain/awp158 PMC2732267

[bib43] Sadun AA Win PH Ross-Cisneros FN Walker SO qCarelli V Leber's hereditary optic neuropathy differentially affects smaller axons in the optic nerve Transact Am Ophthalmol Soc 2000 98 223 235 PMC1298228 11190025

[bib44] Carelli V Rugolo M Sgarbi G Ghelli A Zanna C Baracca A Bioenergetics shapes cellular death pathways in Leber's hereditary optic neuropathy: a model of mitochondrial neurodegeneration Biochim Biophys Acta 2004 1658 (1-2 172 179 15282189 10.1016/j.bbabio.2004.05.009

[bib45] Lin CS Sharpley MS Fan W Waymire KG Sadun AA Carelli V Mouse mtDNA mutant model of Leber hereditary optic neuropathy Proc Natl Acad Sci USA 2012 109 (49 20065 20070 23129651 10.1073/pnas.1217113109 PMC3523873

[bib46] Yu-Wai-Man P Chinnery PF Dominant optic atrophy—Novel OPA1 mutations and revised prevalence estimates Ophthalmology 2013 120 (8 1712 23916084 10.1016/j.ophtha.2013.04.022 PMC6542663

[bib47] Alexander C Votruba M Pesch UEA Thiselton DL Mayer S Moore A Identification of the gene responsible for dominant optic atrophy (OPA1) on chromosome 3q28 Am J Hum Genet 2000 67 (4 40

[bib48] Delettre C Lenaers G Griffoin JM Gigarel N Lorenzo C Belenguer P Nuclear gene OPA1, encoding a mitochondrial dynamin-related protein, is mutated in dominant optic atrophy Nat Genet 2000 26 (2 207 210 11017079 10.1038/79936

[bib49] Ferre M Bonneau D Milea D Chevrollier A Verny C Dollfus H Molecular screening of 980 cases of suspected hereditary optic neuropathy with a report on 77 novel OPA1 mutations Hum Mut 2009 30 (7 E692 E705 19319978 10.1002/humu.21025

[bib50] Marchbank NJ Craig JE Leek JP Toohey M Churchill AJ Markham AF Deletion of the OPA1 gene in a dominant optic atrophy family: evidence that haploinsufficiency is the cause of disease J Med Genet 2002 39 (8 e47 12161614 10.1136/jmg.39.8.e47 PMC1735190

[bib51] Yu-Wai-Man P Griffiths PG Burke A Sellar PW Clarke MP Gnanaraj L The prevalence and natural history of dominant optic atrophy due to OPA1 mutations Ophthalmology 2010 117 (8 1538 15461546.e1. 20417570 10.1016/j.ophtha.2009.12.038 PMC4040407

[bib52] Yu-Wai-Man P Bailie M Atawan A Chinnery PF Griffiths PG Pattern of retinal ganglion cell loss in dominant optic atrophy due to OPA1 mutations Eye 2011 25 (5 597 601 10.1038/eye.2011.2 PMC3094220 21378995

[bib53] Yu-Wai-Man P Griffiths PG Gorman GS Lourenco CM Wright AF Auer-Grumbach M Multi-system neurological disease is common in patients with OPA1 mutations Brain 2010 133 771 786 20157015 10.1093/brain/awq007 PMC2842512

[bib54] Lenaers G Olichon A Delettre C Hamel C Belenguer P Functions and dysfunctions of the human dynamin OPA1 Biochim Biophys Acta 2004 1657 37

[bib55] Chevrollier A Guillet V Loiseau D Gueguen N de Crescenzo M-AP Verny C Hereditary optic neuropathies share a common mitochondrial coupling defect Ann Neurol 2008 63 (6 794 798 18496845 10.1002/ana.21385

[bib56] Zanna C Ghelli A Porcelli AM Karbowski M Youle RJ Schimpf S OPA1 mutations associated with dominant optic atrophy impair oxidative phosphorylation and mitochondrial fusion Brain 2008 131 352 367 18222991 10.1093/brain/awm335

[bib57] Frezza C Cipolat S de Brito OM Micaroni M Beznoussenko GV Rudka T OPA1 controls apoptotic cristae remodeling independently from mitochondrial fusion Cell 2006 126 (1 177 189 16839885 10.1016/j.cell.2006.06.025

[bib58] Kushnareva YE Gerencser AA Bossy B Ju WK White AD Waggoner J Loss of OPA1 disturbs cellular calcium homeostasis and sensitizes for excitotoxicity Cell DeathDiffer 2013 20 (2 353 365 10.1038/cdd.2012.128 PMC3554330 23138851

[bib59] Yu-Wai-Man P Sitarz KS Samuels DC Griffiths PG Reeve AK Bindoff LA OPA1 mutations cause cytochrome c oxidase deficiency due to loss of wild-type mtDNA molecules Hum Mol Genet 2010 19 (15 3043 3052 20484224 10.1093/hmg/ddq209 PMC2901142

[bib60] Bailie M Votruba M Griffiths PG Chinnery PF Yu-Wai-Man P Visual and psychological morbidity among patients with autosomal dominant optic atrophy Acta ophthalmol 2013 91 (5 e413 e414 23452392 10.1111/aos.12077 PMC3798121

[bib61] Pfeffer G Majamaa K Turnbull DM Thorburn D Chinnery PF Treatment for mitochondrial disorders Cochrane Database Syst Rev 2012 4 CD004426 22513923 10.1002/14651858.CD004426.pub3 PMC7201312

[bib62] Pfeffer G Horvath R Klopstock T Mootha VK Suomalainen A Koene S New treatments for mitochondrial disease-no time to drop our standards Nat Rev Neurol 2013 9 (8 474 481 23817350 10.1038/nrneurol.2013.129 PMC4967498

[bib63] Newman NJ Treatment of hereditary optic neuropathies Nat Rev Neurol 2012 8 (10 545 556 22945544 10.1038/nrneurol.2012.167

[bib64] Huang CC Kuo HC Chu CC Kao LY Rapid visual recovery after coenzyme Q10 treatment of Leber Hereditary Optic Neuropathy J Neuro-Ophthalmol 2002 22 (1 66 10.1097/00041327-200203000-00036 11937918

[bib65] Sadun AA Chicani CF Ross-Cisneros FN Barboni P Thoolen M Shrader WD Effect of EPI-743 on the clinical course of the mitochondrial disease Leber hereditary optic neuropathy Arch Neurol 2012 69 (3 331 338 22410442 10.1001/archneurol.2011.2972

[bib66] Mashima Y Hiida Y Oguchi Y Remission of Lebers hereditary optic neuropathy with Idebenone Lancet 1992 340 (8815 368 369 1353825 10.1016/0140-6736(92)91442-b

[bib67] Mashima Y Kigasawa K Wakakura M Oguchi Y Do idebenone and vitamin therapy shorten the time to achieve visual recovery in Leber hereditary optic neuropathy J Neuro-Ophthalmol 2000 20 (3 166 170 10.1097/00041327-200020030-00006 11001192

[bib68] Barnils N Mesa E Munoz S Ferrer-Artola A Arruga J Response to idebenone and multivitamin therapy in Leber's hereditary optic neuropathy Arch de la Sociedad Espanola de Oftalmol 2007 82 (6 377 380 10.4321/s0365-66912007000600012 17573650

[bib69] Klopstock T Metz G Yu-Wai-Man P Buechner B Gallenmueller C Bailie M Persistence of the treatment effect of idebenone in Leber's hereditary optic neuropathy Brain 2013 136 e230 23388409 10.1093/brain/aws279 PMC3572931

[bib70] Carelli V La Morgia C Valentino ML Rizzo G Carbonelli M De Negri AM Idebenone treatment in Leber's hereditary optic neuropathy Brain 2011 134 e188 21810891 10.1093/brain/awr180

[bib71] Limb GA Martin KR Current prospects in optic nerve protection and regeneration: sixth ARVO/Pfizer Ophthalmics Research Institute Conference Invest Ophthalmol Vis Sci 2011 52 (8 5941 5954 21804096 10.1167/iovs.10-6894

[bib72] Newman NJ Biousse V David R Bhatti MT Hamilton SR Farris BK Prophylaxis for second eye involvement in Leber hereditary optic neuropathy: An open-labeled, nonrandomized multicenter trial of topical brimonidine purite Am J Ophthalmol 2005 140 (3 407 415 16083844 10.1016/j.ajo.2005.03.058

[bib73] Thouin A Griffiths PG Hudson G Chinnery PF Yu-Wai-Man P Raised intraocular pressure as a potential risk factor for visual loss in Leber hereditary optic neuropathy Plos ONE 2013 8 (5 e63446 23667621 10.1371/journal.pone.0063446 PMC3646743

[bib74] Wong A Cortopassi G mtDNA Mutations confer cellular sensitivity to oxidant stress that is partially rescued by calcium depletion and cyclosporin A Biochem Biophys Res Commun 1997 239 (1 139 145 9345284 10.1006/bbrc.1997.7443

[bib75] Porcelli AM Angelin A Ghelli A Mariani E Martinuzzi A Carelli V Respiratory complex I dysfunction due to mitochondrial DNA mutations shifts the voltage threshold for opening of the permeability transition pore toward resting levels J Biol Chem 2009 284 (4 2045 2052 19047048 10.1074/jbc.M807321200

[bib76] Malik A Golnik K Hyperbaric oxygen therapy in the treatment of radiation optic neuropathy J Neuro-Ophthalmol 2012 32 (2 128 131 10.1097/WNO.0b013e3182464c88 22286188

[bib77] Desmet KD Paz DA Corry JJ Eells JT Wong-Riley MTT Henry MM Clinical and experimental applications of NIR-LED photobiomodulation Photomed Laser Surg 2006 24 (2 121 128 16706690 10.1089/pho.2006.24.121

[bib78] Barboni P Valentino ML La Morgia C Carbonelli M Savini G De Negri A Idebenone treatment in patients with OPA1-mutant dominant optic atrophy Brain 2013 136 e231 23388408 10.1093/brain/aws280

[bib79] Davies VJ Hollins AJ Piechota MJ Yip W Davies JR White KE Opa1 deficiency in a mouse model of autosomal dominant optic atrophy impairs mitochondrial morphology, optic nerve structure and visual function Hum Mol Genet 2007 16 (11 1307 1318 17428816 10.1093/hmg/ddm079

[bib80] Levin LA Axonal loss and neuroprotection in optic neuropathies Can J Ophthalmol 2007 42 (3 403 408 17508035

[bib81] Pesce V Fracasso F Cassano P Lezza AMS Cantatore P Gadaleta MN Acetyl-L-carnitine supplementation to old rats partially reverts the age-related mitochondrial decay of soleus muscle by activating peroxisome proliferator-activated receptor gamma coactivator-1 alpha-dependent mitochondrial biogenesis Rejuv Res 2010 13 (2-3 148 151 10.1089/rej.2009.0955 20370498

[bib82] Pesce V Nicassio L Fracasso F Musicco C Cantatore P Gadaleta MN Acetyl-L-carnitine activates the peroxisome proliferator-activated receptor-gamma coactivators PGC-1 alpha/PGC-1 beta-dependent signaling cascade of mitochondrial biogenesis and decreases the oxidized peroxiredoxins content in old rat liver Rejuv Res 2012 15 (2 136 139 10.1089/rej.2011.1255 22533417

[bib83] Guy J New therapies for optic neuropathies: development in experimental models Curr Opin Ophthalmol 2000 11 (6 421 429 11141636 10.1097/00055735-200012000-00007

[bib84] Guy J Qi XP Pallotti F Schon EA Manfredi G Carelli V Rescue of a mitochondrial deficiency causing Leber hereditary optic neuropathy Ann Neurol 2002 52 (5 534 542 12402249 10.1002/ana.10354

[bib85] Ellouze S Augustin S Bouaita A Bonnet C Simonutti M Forster V Optimized allotopic expression of the human mitochondrial ND4 prevents blindness in a rat model of mitochondrial dysfunction Am J Hum Genet 2008 83 (3 373 387 18771762 10.1016/j.ajhg.2008.08.013 PMC2556433

[bib86] Guy J Qi X Koilkonda RD Arguello T Chou T-H Ruggeri M Efficiency and safety of AAV-mediated gene delivery of the human ND4 complex I subunit in the mouse visual system Invest Ophthalmol Vis Sci 2009 50 (9 4205 4214 19387075 10.1167/iovs.08-3214 PMC3085487

[bib87] Qi X Sun L Hauswirth WW Lewin AS Guy J Use of mitochondrial antioxidant defenses for rescue of cells with a Leber hereditary optic neuropathy-causing mutation Arch Ophthalmol 2007 125 (2 268 272 17296905 10.1001/archopht.125.2.268

[bib88] Seo BB Nakamaru-Ogiso E Flotte TR Matsuno-Yagi A Yagi T *In vivo* complementation of complex I by the yeast Ndi1 enzyme—Possible application for treatment of Parkinson disease J Biol Chem 2006 281 (20 14250 14255 16543240 10.1074/jbc.M600922200

[bib89] Iyer S Bergquist K Young K Gnaiger E Rao RR Bennett JP Jr Mitochondrial gene therapy improves respiration, biogenesis, and transcription in G11778A Leber's hereditary optic neuropathy and T8993G Leigh's syndrome cells Hum Gene Ther 2012 23 (6 647 657 22390282 10.1089/hum.2011.177 PMC3392617

[bib90] Yu H Koilkonda RD Chou T-H Porciatti V Ozdemir SS Chiodo V Gene delivery to mitochondria by targeting modified adenoassociated virus suppresses Leber's hereditary optic neuropathy in a mouse model Proc Natl Acad Sci USA 2012 109 (20 E1238 E1247 22523243 10.1073/pnas.1119577109 PMC3356643

[bib91] Yu H Mehta A Wang G Hauswirth WW Chiodo V Boye SL Next-generation sequencing of mitochondrial targeted AAV transfer of human ND4 in mice Mol Vision 2013 19 1482 1491 PMC3712668 23869167

[bib92] Sarzi E Angebault C Seveno M Gueguen N Chaix B Bielicki G The human OPA1(delTTAG) mutation induces premature age-related systemic neurodegeneration in mouse Brain 2012 135 3599 3613 23250881 10.1093/brain/aws303

[bib93] Marchetti V Krohne TU Friedlander DF Friedlander M Stemming vision loss with stem cells J Clin Invest 2010 120 (9 3012 3021 20811157 10.1172/JCI42951 PMC2929728

[bib94] Dahlmann-Noor A Vijay S Jayaram H Limb A Khaw PT Current approaches and future prospects for stem cell rescue and regeneration of the retina and optic nerve Can J Ophthalmol 2010 45 (4 333 341 20648090 10.3129/i10-077

[bib95] Johnson TV Bull ND Hunt DP Marina N Tomarev SI Martin KR Neuroprotective effects of intravitreal mesenchymal stem cell transplantation in experimental glaucoma Invest Ophthalmol Vis Sci 2010 51 (4 2051 2059 19933193 10.1167/iovs.09-4509 PMC2868400

[bib96] Connick P Kolappan M Crawley C Webber DJ Patani R Michell AW Autologous mesenchymal stem cells for the treatment of secondary progressive multiple sclerosis: an open-label phase 2a proof-of-concept study Lancet Neurol 2012 11 (2 150 156 22236384 10.1016/S1474-4422(11)70305-2 PMC3279697

[bib97] Craven L Elson JL Irving L Tuppen HA Lister LM Greggains GD Mitochondrial DNA disease: new options for prevention Hum Mol Genet 2011 20 R168 R174 21852248 10.1093/hmg/ddr373 PMC3179382

[bib98] Craven L Tuppen HA Greggains GD Harbottle SJ Murphy JL Cree LM Pronuclear transfer in human embryos to prevent transmission of mitochondrial DNA disease Nature 2010 465 (7294 U82 U89 10.1038/nature08958 PMC2875160 20393463

[bib99] Tachibana M Sparman M Sritanaudomchai H Ma H Clepper L Woodward J Mitochondrial gene replacement in primate offspring and embryonic stem cells Nature 2009 461 (7262 367 372 19710649 10.1038/nature08368 PMC2774772

[bib100] Tachibana M Amato P Sparman M Woodward J Sanchis DM Ma H Towards germline gene therapy of inherited mitochondrial diseases Nature 2013 493 (7434 627 631 23103867 10.1038/nature11647 PMC3561483

